# The Exocrine Chemistry of the Parasitic Wasp *Sphecophaga orientalis* and Its Host *Vespa orientalis*: A Case of Chemical Deception?

**DOI:** 10.3390/insects12010002

**Published:** 2020-12-23

**Authors:** Shahar Dubiner, Nitzan Cohen, Mika Volov, Abraham Hefetz, Rya Seltzer, Eran Levin

**Affiliations:** 1 School of Zoology, Faculty of Life Sciences, Tel Aviv University, 6997801 Tel Aviv, Israel; dubiner@mail.tau.ac.il (S.D.); cknitzan@gmail.com (N.C.); mikavolov@mail.tau.ac.il (M.V.); hefetz@tauex.tau.ac.il (A.H.); ryaseltz@mail.tau.ac.il (R.S.); 2 Faculty of Marine Science, Ruppin Academic Center, 4025000 Michmoret, Israel

**Keywords:** *Vespa*, *Sphecophaga*, parasitoid, CHC, oleic acid, rose oxide

## Abstract

**Simple Summary:**

The wasp *Sphecophaga orientalis* is a parasitoid of the Oriental hornet (*Vespa orientalis*) in its subterranean colonies. We describe this parasitoid’s occurrence in hornet nests in Israel and compare the chemical composition of cuticular washes of both species. The dissimilarity between the two excludes the possibility that the parasite uses either camouflage or chemical mimicry to evade host aggression. Because the parasitoid features large amounts of the necrophoric compound oleic acid, we suggest that, due to this compound, the host considers the parasite as refuse and ignores its presence. The parasitoid head also contains rose oxide, a repellent, possibly used to repel aggressive workers and which, combined with its necrophoric odor, enables it to remain in the nest undisturbed.

**Abstract:**

The main challenge facing a parasite of social insects lies in deceiving its host’s detection and defense systems in order to enter and survive within the host colony. *Sphecophaga orientalis* is an ichneumonid wasp that parasitizes the pupae of the Oriental hornet *Vespa orientalis*. In Israel’s Mediterranean region, this parasitoid infects on average 23.48% (8–56%) of the host pupal cells. Observation of colonies brought to the laboratory revealed that the parasite moves around within the colony without being aggressed by the host workers. To assess how the parasite evades host detection and defense, we compared the cuticular hydrocarbon (CHC) profiles of both species. There was little similarity between the parasite and the host workers’ CHC, refuting the hypothesis of chemical mimicry. The parasite’s CHCs were dominated by linear alkanes and alkenes with negligible amounts of branched alkanes, while the host workers’ CHCs were rich in branched alkanes and with little or no alkenes. Moreover, the parasite cuticular wash was markedly rich in oleic acid, previously reported as a cue eliciting necrophoric behavior. Since nests of Oriental hornets are typified by large amounts of prey residues, we suggest that, due to its unfamiliar CHCs and the abundance of oleic acid, the parasite is considered as refuse by the host. We also detected rose oxide in the parasitoid head extracts. Rose oxide is a known insect repellent, and can be used to repel and mitigate aggression in workers. These two factors, in concert, are believed to aid the parasite to evade host aggression.

## 1. Introduction

Social hymenopterans establish colonies, sometimes containing thousands of individuals from different castes and life stages. Living in a social group of kin at a high population density poses many inherent challenges. Social insect colonies constitute a rich resource and, therefore, are vulnerable to parasite and predator attack. Consequently, social species have evolved a sophisticated nestmate recognition system, which is chemically driven in most species, for differentiating between nestmates and alien invaders [1,2]. Research has shown that this ability is commonly based on the cuticular hydrocarbons’ (CHC) chemical profile on the insects’ bodies [3,4,5,6]. CHCs compose the waxy outermost layer of the insect’s cuticle, reducing evaporative water loss. However, the accessibility and high variability of CHCs make them a valuable cue for recognition by friends and foe alike. In general, social insects have developed this chemical profile into a reliable nestmate recognition signal [4,5], presenting an obstacle that potential invaders must find ways to overcome [6].

The hymenopteran broods are usually immobile and cannot defend themselves, making them a desirable target for predators or parasites [7]. Some predators invade the nest to consume the brood, such as ants that invade wasp nests [8]; while in other cases, individuals of the same or related species invade the nest to kidnap the brood and obtain a new workforce (e.g., slave-making ants [9,10]). The nest, therefore, requires effective protection, and social insects achieve this by various means. For example, in many species the nest entrance is guarded, preventing predators and parasites from entering the colony [11,12]; social insects often construct nests with narrow entrances that can be more easily guarded [13,14]. Furthermore, many social insect workers are equipped with chemical and mechanical weapons they can employ against intruders, even to the point of self-sacrifice [15,16].

One of the defense mechanisms employed by social insects against intruders and parasitism is that of species and nestmate recognition. Consequently, parasites and invertebrate-predators must avoid host detection in order to exploit their target resource. How such parasites deceive their hosts and penetrate the colony, and sometimes complete their entire life cycle in their host’s nest, has been the subject of many studies and involves various strategies. The three main strategies used by such parasites are: chemical mimicry, chemical insignificance or camouflage, and appeasement/repellent substances [11]. Behavioral invasive strategies are also common, such as vigilant behavior [17] and flattening to the ground [18].

A significant portion of all known insects are parasitoids (about 10%), most of which are wasps [19]. *Sphecophaga orientalis* (Donovan) (Hymenoptera: Ichneumonidae) is a hymenopteran parasitoid that exploits *Vespa orientalis* (Linnaeus) colonies in the Middle East. Most of its biology is known from studies of its close relative species *S. vesparum* (Curtis)*,* whose biology is very similar; indeed, until recently [20] they were considered to be the same species. *S. vesparum* exploits vespid colonies in temperate climates [21,22], laying its eggs on the newly pupated individuals, through the silk cap. *S. vesparum* reproduce by gynogenesis (females can reproduce without sexual fertilization), and males are usually absent. There are two adult forms of *S. vesparum*: brachypterous females with limited flight ability, which emerge from white cocoons and remain to reproduce within the nest; and winged females, which emerge from yellow cocoons, overwinter in the nest, and disperse [21]. *S*. *vesparum* is a specific parasitoid to the subfamily Vespinae [20]. In a recent comparative study, the authors compared the cuticular hydrocarbons (CHC) of *S. vesparum* and two of its host species (*Vespula germanica* and *Vespula vulgaris*). A partial congruence, especially in linear alkanes, of the CHCs of the parasitoid and its hosts, was argued as an indication of “partial chemical mimicry” that plays a role in the parasite’s ability to remain undetected in the colony [22].

In a previous study, *S*. *vesparum* was found in a large proportion of Oriental hornet (*V. orientalis*) colonies in semi-arid sandy habitats in southwest Israel. Within these nests, 2.5–95.1% of host cells were parasitized [23]. This parasite’s taxonomic status was later revised, and based on genital morphology and genetic variance, it was reclassified as a new species: *S. orientalis* [24]. The host of the parasitoid, the Oriental hornet, is a true medium-size hornet (~250 mg) with a wide distribution covering North Africa, southern Asia, and southern Europe, and it has recently invaded North America [25]. Oriental hornets establish annual colonies in the spring, usually in underground cavities, that can comprise up to several thousand individuals by the end of summer [26,27]. The Oriental hornet is adapted to aridity and is the only member of the genus *Vespa* found in arid and warm habitats [28]. In Israel, it is found throughout the country, even in the extreme desert. This hornet is considered a significant agricultural pest, attacking honeybee hives (*Apis melifera*) and damaging sugary summer fruits like grapes, dates, figs, and pomegranates [25,29,30].

**Figure 1:**
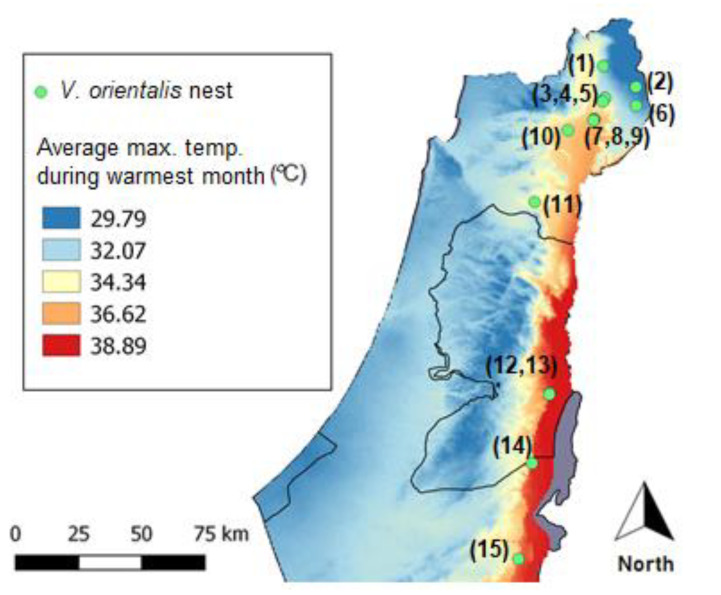
Oriental hornet collection sites shown on an annual mean temperature map. Nests 1–14 collected during 2018; nest 15 collected during 2019.

We recorded parasitism by *S. orientalis* (a species very closely related to *S*. *vesparum*) of the Oriental hornet (*Vespa orientalis*) throughout its distribution range in Israel, in both the Mediterranean and desert habitats (Figure 1). Here, we examined the parasitization rate and cuticular hydrocarbon (hereafter, CHC) profiles of both host and parasitoid to test the hypothesis that this parasitoid uses chemical mimicry to deceive its vespid host. Our findings reject the chemical mimicry hypothesis [22]. Instead, we posit three alternative strategies that, in concert, enable *S. orientalis* to penetrate and deceive its host, *V. orientalis*.

## 2. Material and Methods

### 2.1. Collection of Hornets

We collected 15 colonies of *V. orientalis* in Israel during the end of the season, when sexuals are produced (September–November), from both Mediterranean (*n* = 11) and arid, warm desert habitats (*n* = 4, Figure 1 and Table S1). Nests were located by following workers from water resources back to their nest. Nests were excavated whole and brought to the laboratory, where combs of each colony containing only the pupae (larvae were removed in the lab) were placed individually in cardboard boxes and kept at ~25 °C. Newly enclosed individuals of *V. orientalis* and female *S. orientalis* were collected daily, placed in individual tubes, frozen, and stored at –80 °C for several weeks until being used for CHC extraction. All collections were carried out under permit 2019/42252 from the Israel Nature and Parks Protection Authority. 

### 2.2. Parasitization Analysis 

We counted the number of parasitized cells in each comb, considering both empty and inhabited parasitoid cocoons as positive for parasitization. For each nest, we summarized the findings, incorporating the total number of cells, cell size (indicating caste), rate of parasitization (regarding both the number of cocoons per total cell number and the percentage of cells infested), and yellow-to-white (winged or brachypterous forms, respectively) parasitoid cocoon ratio.

### 2.3. Cuticular Hydrocarbon Analysis

From each of four of the infested nests, chosen from different regions (4, 7, 10, and 11; see Figure 1), we randomly sampled nine *S. orientalis* winged females and nine *V. orientalis* workers. Extractions for chemical analyses were performed immediately after removing the specimens from the freezer to ensure their freshness. We extracted the cuticular hydrocarbons as follows: we separated the body parts into head, thorax, and abdomen. Thorax and abdomen were immersed in hexane for 20 min, and heads were immersed in dichloromethane for over two days to allow polar compound extraction. *V. orientalis* was extracted individually, but the smaller *S. orientalis* wasps were pooled in groups of three to obtain stronger signals in the chromatograms. Throughout our study, and in all the collected nests, only the winged forms of *S. orientalis* were found and used for analysis. Initial CHC analyses were performed by GC/MS (Agilent 5977) equipped with a DB-5MS UI column. The GC injector was maintained at 250 °C and the oven temperature was programmed from 60 (1 min hold) to 300 °C at 10 °C/min and held at the final temperature for 15 min (total run time 40 min). The ionization mode was electron impact (70 eV). The mass selective detector was operated in the scan mode between 40 and 600 AMU. Compounds were identified by comparison with mass spectra available on an NIST database and by mass fragmentation—see Table S3 for details. 

For quantitative analyses, samples were analyzed in a Varian-3900XL gas chromatograph (GC-FID) equipped with a silica column (Varian, VF-5ms, 30 m × 0.25 m, DF = 0.25). Samples were run under the same temperature program as above. CHCs were identified by retention times and compared with those obtained in the GC/MS run and with synthetic hydrocarbons. To ensure proper identification, the same sample was sometimes also run by both GC and GC/MS. Relative compound quantification was achieved by peak integration using Galaxie 1.9 software.

### 2.4. Statistical Analysis

We used a stepwise discriminant analysis (DA) to detect differences in CHC profiles between different *V. orientalis* colonies and (separately) their parasitoids. We used this analysis of CHC profiles to determine which of the components might contribute to the discrimination between nests: in each step of the analysis, the variable (CHC) entered was the one with the highest F to Enter value, being that which maximizes the discrimination between the four groups (colonies). Variables with an F to Enter value lower than the entry criteria (3.48) were removed from the analysis. The analysis was conducted using SPSS software (Version 25, SPSS Inc., Chicago, IL, USA).

## 3. Results

### 3.1. Parasitization Analysis

Of the 14 nests from the Mediterranean, all but one (colony 2) and none from the extreme arid zone (colonies 12, 13, 14 and 15) had been parasitized by *S. orientalis*, suggesting a limit to its distribution into an arid region. In the parasitized nests, the mean percentage of total infected cells was 23.48%: 30.25 ± 14.98% (SD) for queens (identified by cell size) and 17.96 ± 17.55% (SD) for workers or males (indistinguishable by cell size), but this difference was not statistically significant (Kruskal–Wallis: *H* = 3.277, *p* = 0.07). The mean number of cocoons of *S. orientalis* per parasitized cell was 2.24 ± 0.21 for queen pupae and 2.02 ± 0.23 for workers and males (see Table S2). The highest number of parasitoids was documented in nest #10, which contained a total of 11 parasitoids per single queen cell, all of which were able to feed and develop from a single host pupa. 

### 3.2. Cuticular Analysis

**Figure 2:**
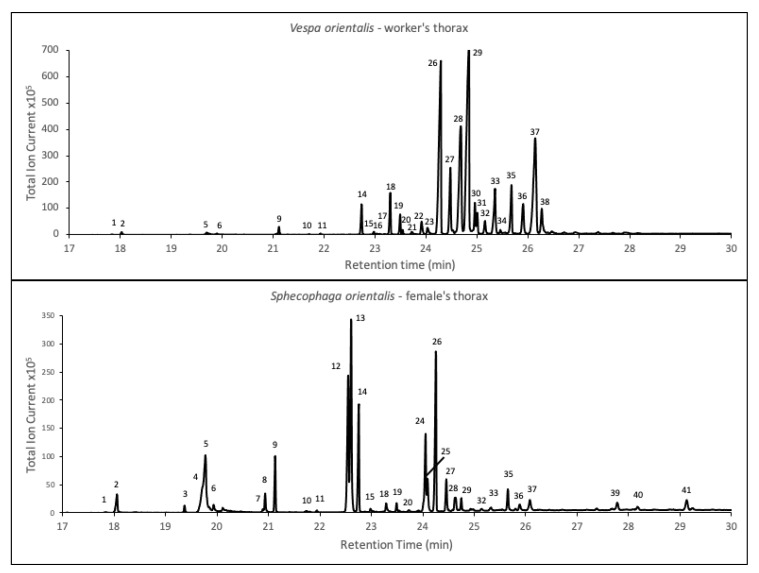
GC chromatograms of thoracic cuticular extracts from three adult *S. orientalis* (**top**) and a single *V. orientalis* (**bottom**). Component numbers correspond to the specific compounds, diagnostic ions, and relative amounts in Table S3.

**Figure 3:**
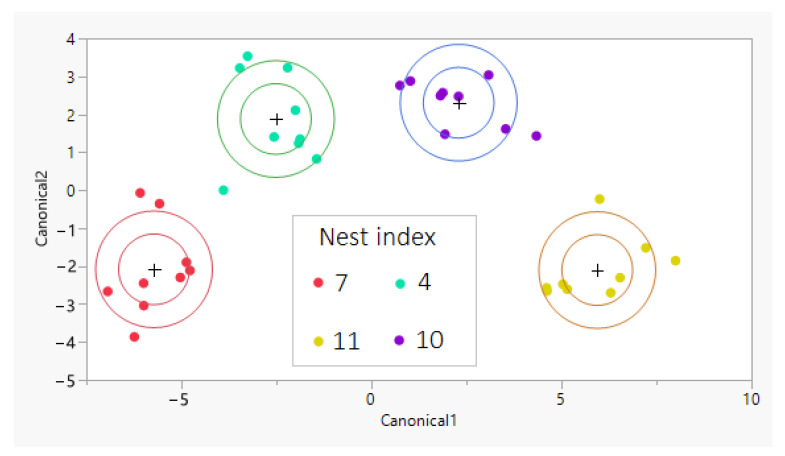
Canonical plot for the discriminant analysis for thoracic cuticular hydrocarbons (CHCs) of *V. orientalis* from four infested nests (see Figure 1 for their locations). Discrimination by CHCs was significant (Wilks’s λ = 0.005, χ^2^ = 146.661, *p* < 0.001). Circles represent the 50% normal contours (inner) and 95% confidence level for means (outer).

Chemical analysis of the cuticular washes of *V. orientalis* revealed a mixture of linear and branched alkanes (Figure 2 and Table S3). The linear alkanes ranged from tricosane to triacontane, with heptacosane dominating. Branched alkanes were particularly abundant in the C27 complex, comprising a mix of 11- and 13-methylheptacosane, 11,15 dimethylheptacosane and 3-methyl heptacosane. There was a lesser proportion of branched alkanes in the C29 complex, with the exception of 13,15 dimethylnonacosane, which was present in relatively large proportions. Discriminant analyses of workers’ thoracic CHCs showed nest-specific composition (Figure 3). The discriminant model was significant (step 1: Wilks’s λ = 0.133, F = 63.226, *p* < 0.001; step 2: Wilks’s λ = 0.016, F = 65.112, *p* < 0.001), with significant difference between colonies in the mean of Functions 1 through 3 (Wilks’s λ = 0.005, χ^2^ = 146.661, *p* < 0.001). Nest specificity was also significant for abdominal samples (Wilks’s λ = 0.003, χ^2^ = 168.718, *p* < 0.001), but note that those samples possibly include the content of abdominal glands that are absent in the thorax. Stepwise DA found the number of hydrocarbons discriminating between colonies (i.e., those with *F* statistic higher than criteria to enter into the stepwise analysis) to be five in the thorax samples and seven in the abdomen samples (see Table 1). These are the ones that are most varied between nests while they are conserved within each nest, and therefore are the most likely to be used as recognition cues. Nearly all of them are branched-alkanes (mono- and dimethyl alkanes).

**Figure 4:**
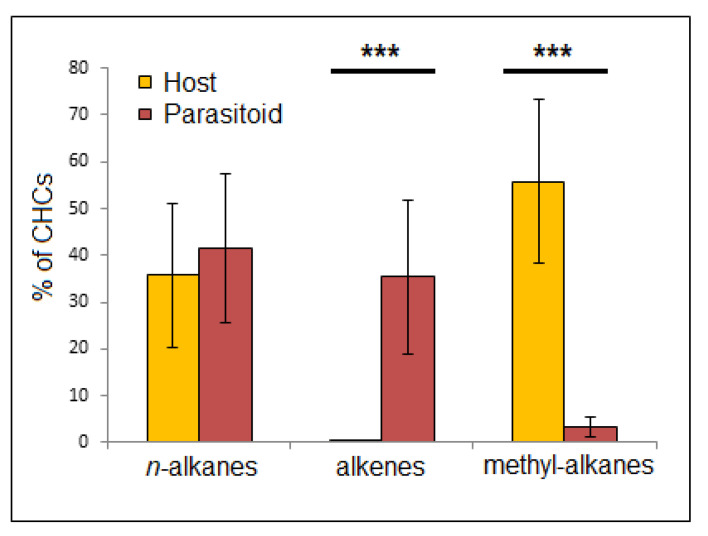
Comparison between host and parasitoid for relative content of total *n*-alkanes, alkenes, and methyl alkanes. *** indicates *p*-value < 0.001.

The cuticular washes of *S. orientalis* were typified, in contrast to those of its host, *V. orientalis*, by an abundance of alkenes, of which two isomers of pentacosane dominated— 42% alkenes in the parasite compared to 0.04% in the host (Figure 2 and Table S3). In contrast, branched alkanes that were pronounced in the host (59.7% of total CHCs) were found only in minute amounts in the parasite (2.4% of total CHC). Other than the one alkane (heptacosane) of the CHCs that contributed to the discrimination between hornet nests in the DA, nearly all other alkanes were absent, and none contributed more than 1% of the parasitoid’s total CHCs. Kruskal–Wallis test showed these differences to be highly significant (*p* << 0.001 for methyl-alkanes, *p* << 0.001 for alkenes, but *p* = 0.167 for alkanes; see Figure 4).

Discriminant analysis for parasitoid CHCs also yielded significant discrimination among colonies (Wilks’s λ < 0.001, χ^2^ = 77.061, *p* < 0.001). Note that in this analysis, in contrast to the individually tested hosts, each sample of parasitoids (*n* = 12) was a pool of three individuals.

In addition to hydrocarbons, high levels of oleic acid were found in all *S. orientalis* samples (mean 16.13 ± 5.34% of total thoracic GC profile, compared to 3.12 ± 2.81% in the host; Figure 2, Table S3). Head extracts also contained the volatile pyran-monoterpene rose oxide ((4R)-2-(2-Methyl-1-propenyl)-4-methyltetrahydropyran), although in relatively small amounts (<1% of GC peak area). The identity of rose oxide was validated using its synthetic standard (Sigma–Aldrich, Rehovot, Israel).

## 4. Discussion

We found high infestation rates of *V. orientalis* nests by the parasitoid *S. orientalis*, ranging from 8 to 56% of the cells per colony. At the end of summer, parasitoids were so abundant in the infested hornet colonies that almost no viable hornet pupae remained in these colonies. All but one of the nests collected from the Mediterranean region were parasitized, while none of the four nests from the arid region were parasitized. We thus suggest that a warm and dry climate may restrict *S. orientalis* distribution.

One of the obstacles that a parasite of social insects must overcome is that of infiltrating the colony, which is generally well guarded by the workers. Once inside the nest, the parasite needs to move unnoticed while laying its eggs. Likewise, the newly enclosed parasites must survive long enough within the nest before being able to disperse. One option by which to remain unnoticed or unchallenged is that of chemical mimicry, whereby the parasites emit a chemical signature that is congruent with that of its host and renders it “invisible” within the nest. Contrary to recent findings of partial mimicry in a closelyrelated species, *S. vesparum* [22], we found no evidence of host chemical mimicry in *S. orientalis*. Qualitatively, the host and its parasite CHC profiles are utterly different (Figure 2, Table S1), refuting the hypothesis of chemical mimicry or chemical camouflage. Another option for avoiding notice is that of the parasite possessing very low CHC quantities, to the point of being chemically insignificant [2,11,31]. However, this explanation too does not seem to hold here, as the parasitoid’s CHC levels are high enough to be easily detected by the host. The third possible explanation—chemical transparency [32]—may offer the best explanation here, at least in part. A comparison of the CHCs of *V. orientalis* and *S. orientalis* revealed that only the alkanes are common to both host and parasitoid. Alkanes, however, have been suggested to play an inferior role in recognition between colonies or even species of social insects [33,34,35,36,37,38]. In two other wasp species, the methyl alkanes were suggested to present nestmate recognition cues [35,39], and their practical absence from the parasitoid *S. orientalis* CHCs lends credence to this hypothesis. Our discriminant analysis for *V. orientalis*, which revealed methyl alkanes as the main components used for discriminating between nests, supports that hypothesis. Martin et al. [32] obtained somewhat similar results when studying the eggs of *V. dybowskii*, a social parasite of the hornets *V. simillima* and *V. crabro*. They suggested that if branched-alkanes are indeed the main component of vespid nestmate recognition, these specific compounds’ minor levels may reduce detectability. This same concept of chemical transparency, albeit pertaining to a very different aspect of parasitization, may explain and support this complete lack of overlap between host and parasitoid CHCs. That said, without behavioral assays to confirm this we cannot rule out that parasitoid CHCs in fact lack adaptations to the host, and the described effect may be coincidental or irrelevant. While the discriminant analysis between parasitoids was also significant and reflected different CHCs, this is less likely to be an adaptation of the different colonies and more probably a passive result of relatedness or environment.

We suggest that the parasite takes advantage of the hosts’ treatment of decaying prey brought back to the nest by the workers. Nests of *V. orientalis* are usually littered with decaying arthropod prey [40], thus encountering a carcass covered in CHCs is not infrequent. We can also assume that the CHCs of these carcasses (e.g., honeybees) are markedly different from the specific CHC composition of the colony workers—for example containing high levels of alkenes [41]. Moreover, insect carcasses emit oleic acid, which is known to be a universal “death indicator” in insects [42,43], and is released from the corpse shortly after death, reaching high levels within hours, and remaining so for several days [44].

The presence of high amounts of oleic acid in the parasite (16.13 ± 5.34% of total), but not the host workers, may indicate a complementary strategy for evading host worker aggression. Social insects’ responses to oleic acid are expressed through different behavioral repertoires. Some social insects use this signal to remove dead individuals from the nest, which stimulates hygienic behavior in honeybees and necrophoric behavior in ants [42,45]. Moreover, social insects that scavenge use oleic acid as a cue to recognize dead prey while foraging. However, this behavioral repertoire is not straightforward and it has been demonstrated that this response varies, depending on caste, timing, and context [46,47]. An original study by Hughes et al. suggested that some plant seeds’ elaiosomes demonstrate partial chemical mimicry of dead prey fatty acid components, including oleic acid. They demonstrated that these elaiosome-bearing seeds’ chemical profiles attract omnivorous and carnivorous ants and induce them to collect and disperse the seeds [48]. Using oleic acid as a cue exemplifies a secondary evolutionary development that takes advantage of a characteristic behavior of response to a reliable chemical signal. We hypothesize that a similar evolutionary pattern may govern the interactions between *V. orientalis* and *S. orientalis*. The emission of oleic acid by the parasite, together with the “transparency” of its CHC composition, may represent a novel parasitization strategy in insects: the parasites chemically disguise themselves as the remains of their hosts’ prey. This previously overlooked strategy may be exploiting a suggested universal signal and moreover, may also be utilized by other species.

Nest infiltration strategies employed by parasites are generally those of “sneaking in and if detected repel” [49,50]. We found the monoterpene compound, rose oxide, in the heads of *S. orientalis*. This volatile compound is a component of beetles’ defensive secretion glands [51], and functions as a highly effective repellent and even narcotizing agent [52]. This would make it an excellent repellent for insects in a closed space, such as the cavity of a hornet nest. The use of rose oxide as a repellent by the parasitoid may represent another line of defense in the closed nest cavity, despite its small quantities.

In conclusion, we suggest that the combination of mimicking decaying prey, together with the possible use of rose oxide as a repellent, while each insufficient on its own, might together enable *S. orientalis* to infiltrate and infest host nests with considerable success throughout its range of distribution. We have inferred this possibility indirectly, from examining the presence and abundances of known chemicals in the two species’ cuticular profile, based on the literature and paired with our own observations of *S. orientalis* thriving unmolested in great numbers inside most colonies of *V. orientalis*. However, behavioral assays are still needed in order to test each hypothesis alone and in combination, as well as to assess the relative importance and efficiency of each of the indicated strategies.

## Figures and Tables

**Table 1:** Variables entered into the stepwise discriminant analysis, by order of steps, for both thoracic and abdominal hornet CHCs. Note that all but *n*-C27 are methyl alkanes. Five CHCs were sufficient for discrimination between nests in thoracic samples, and seven in the abdominal samples.

Step	Thorax	Abdomen
1	13,15-dimethyl-C29	2-methyl-C26
2	11- and 13-methyl-C29	13,15-dimethyl-C29
3	3-methyl-C29	11,15-dimethyl-C27
4	12,16-dimethyl-C28	n-C27
5	-	11- and 13-methyl-C27
6	-	4-methyl-C26

## Supplementary Materials

The following are available online at https://www.mdpi.com/2075-4450/12/1/2/s1, Table S1: Coordinates and altitudes of the 15 *V. orientalis* nests collected, nest index indicates location from north to south, Table S2: Summary of parasitization rate of all nests shown (separately for queen cells and worker/drone cells) as a percentage of cells infected and as parasitoid cocoons per total cell number. Nest size and percentage of yellow (overwintering) cocoons are shown, Table S3: Chromatograms and compounds identified by GC/MS for host and parasitoid thorax and abdomen samples. Minor compounds (those found only in some specimens or representing less than 0.5% of the total profile of both species) do not appear in the chromatogram and are not entered into this Table.
