# Supplementary material for: The Exocrine Chemistry of the Parasitic Wasp Sphecophaga orientalis and Its Host Vespa orientalis: A Case of Chemical Deception?
Source: Insects. 2020 Dec 23;12(1):2. doi: 10.3390/insects12010002 (PMC7822126; doi:10.3390/insects12010002)
Supplement: Supplementary file 1 [file insects-12-00002-s001.zip › supplementary-xml/Table S1.pdf]

**Table S1.** Coordinates and altitudes of the 15 *V. orientalis* nests collected, nest index indicates location from north to south.

| Nest Index | Location | Latitude | Longitude | Altitude | Date Collected | Mean Annual Temp. | Mean Precipitation |
|---|---|---|---|---|---|---|---|
| **1** | Kela Alon | 33.13244 | 35.68335 | 651 | 15.10.18 | 21 °C | 500 mm/year |
| **2** | Alonei haBashan | 33.04541 | 35.82088 | 898 | 15.10.18 | 15 °C | 830 mm/year |
| **3** | Ein Fahura 1 | 32.99932 | 35.69105 | 370 | 30.10.18 | 20 °C | 510 mm/year |
| **4** | Ein Fahura 2 | 32.99874 | 35.69191 | 337 | 30.10.18 | 20 °C | 510 mm/year |
| **5** | Ein Shekef | 32.98357 | 35.6824 | 285 | 16.10.18 | 20 °C | 510 mm/year |
| **6** | Einot Peham | 32.96672 | 35.82159 | 704 | 15.10.18 | 18 °C | 630 mm/year |
| **7** | Meshushim Terasa | 32.90979 | 35.64705 | −129 | 6.11.18 | 23 °C | 490 mm/year |
| **8** | Meshushim Bunker | 32.90787 | 35.64453 | −143 | 6.11.18 | 23 °C | 490 mm/year |
| **9** | Meshushim south | 32.90515 | 35.64282 | −153 | 16.10.18 | 23 °C | 490 mm/year |
| **10** | Hukuk | 32.8633 | 35.5366 | −181 | 30.10.18 | 22 °C | 440 mm/year |
| **11** | Ein Harod | 32.56251 | 35.39703 | 91 | 28.10.18 | 19 °C | 420 mm/year |
| **12** | Kalya north | 31.76072 | 35.46002 | −263 | 21.10.18 | 26 °C | 90 mm/year |
| **13** | Kalya south | 31.75538 | 35.45881 | −268 | 21.10.18 | 26 °C | 90 mm/year |
| **14** | Ein Gedi | 31.46627 | 35.38651 | −175 | 22.10.18 | 25 °C | 40 mm/year |
| **15** | Ashalim | 31.06488 | 35.32998 | −188 | 22.9.19 | 25 °C | 40 mm/year |
