# Supplementary material for: The Exocrine Chemistry of the Parasitic Wasp Sphecophaga orientalis and Its Host Vespa orientalis: A Case of Chemical Deception?
Source: Insects. 2020 Dec 23;12(1):2. doi: 10.3390/insects12010002 (PMC7822126; doi:10.3390/insects12010002)
Supplement: Supplementary file 1 [file insects-12-00002-s001.zip › supplementary-xml/Table S2.pdf]

Table S2. Summary of parasitization rate of all nests shown (separately for queen cells and worker/drone cells) as a percentage of cells infected and as parasitoid cocoons per total cell number. Nest size and percentage of yellow (overwintering) cocoons are shown.

**Nest_Index**			QUEENS		
	**#Cells**	**#Cocoon/Parasited Cell**	**%Parasited Cells**	**%Y_Cocoons**	**#Cocoon/Cell#**
**1**	1090	2.1	16.48	65.08	0.12
**2**	uncounted		0		
**3**	0		0		
**4**	420	2.51	44.76	66.53	1.12
**5**	459	2.19	42.7	50.58	0.93
**6**	307	2.12	32.25	48.1	0.68
**7**	1083	1.82	16.07	78.48	0.29
**8**	372	2.21	50.81	53.11	1.12
**9**	1754	2.41	21.44	59.16	0.52
**10**	1110	2.43	38.92	50.1	0.94
**11**	1545	2.35	8.8	80.25	0.21
**12**	0		0		
**13**	uncounted		0		
**14**	0		0		
**15**	uncounted		0		
**mean**	**0**	**2.24**	**30.25**	**61.26**	**0.66**
SD	0	0.21	14.98	12.16	0.39
			OTHERS		
**Nest_index**	**#Cells**	**#Cocoon/Parasited Cell**	**%Parasited Cells**	**%Y_Cocoons**	**#Cocoon/Cell#**
**1**	908	1.69	21.59	43.2	0.45
**2**	uncounted		0		
**3**	1311	2.29	13.65	51.22	0.31
**4**	943	2.04	35.42	63.53	0.72
**5**	0		0		
**6**	632	1.96	7.44	61.96	0.28
**7**	3456	1.61	4.02	37.05	0.06
**8**	651	2.21	56.22	47.34	1.24
**9**	2000	2.14	13.4	54.44	0.29
**10**	1701	2.18	5.29	29.59	0.12
**11**	1227	2.09	4.65	53.78	0.1
**12**	1844		0		
**13**	uncounted		0		
**14**	1195		0		
**15**	uncounted		0		
**mean**	**0**	**2.02**	**17.96**	**49.12**	**0.4**
SD	0	0.23	17.55	11.14	0.38
