# Supplementary material for: The Exocrine Chemistry of the Parasitic Wasp Sphecophaga orientalis and Its Host Vespa orientalis: A Case of Chemical Deception?
Source: Insects. 2020 Dec 23;12(1):2. doi: 10.3390/insects12010002 (PMC7822126; doi:10.3390/insects12010002)
Supplement: Supplementary file 1 [file insects-12-00002-s001.zip › supplementary-xml/Table S3.pdf]

Table S3. Diagnostic ions, retention times, and mean abundances for cuticular compounds in the thorax samples of *V. orientalis* and *S. orientalis*. Chromatograms shown below.

#	Compound	RT	Diagnostic Ions	*Vespa orientalis*		*Sphecophaga orientalis*	
				Mean%	SD	Mean%	SD
1	Palmitoleic acid	17.8	254, 236, 194, 69, 60	0.70	0.62	0.48	0.18
2	Palmitic acid	18	256, 213, 129, 73, 60	0.87	0.49	2.78	0.91
3	Heneicosane	19.4	196, 85, 71, 57, 43	0.10	0.04	1.68	0.68
4	Linoleic acid	19.7	280, 109, 95, 81, 67, 60	0.50	0.50	0.31	0.16
5	Oleic acid	19.8	282, 264, 222, 95, 83, 69, 60	3.12	2.81	16.70	5.66
6	Stearic acid	19.9	284, 241, 185, 129, 73, 60	2.63	2.07	1.33	1.06
7	Tricosene (I isomer)	20.9	322, 125, 97, 83, 69, 55	0.02	0.01	2.94	0.67
8	Tricosene (II isomer)	20.9	322, 125, 97, 83, 69, 55	0.04	0.04	1.90	0.67
9	Tricosane	21.1	324, 99, 85, 71, 57	1.16	0.38	14.58	3.79
10	Tetracosene	21.7	226, 125, 111, 97, 83, 69, 55	0.14	0.07	0	
11	Tetracosane	21.9	338, 99, 85, 71, 57	0.24	0.08	<0.01	
12	Pentacosene (I isomer)	22.5	350, 125, 111, 97, 83, 69, 55	0		18.43	8.62
13	Pentacosene (II isomer)	22.6	350, 125, 111, 97, 83, 69, 55				
14	Pentacosane	22.7	352, 99, 85, 71, 57	4.08	1.26	10.06	1.79
15	11- and 13-methylpentacosane	23	168–224 + 196 + 196	0.22	0.05	0.21	0.07
16	7- methylpentacosane	23.1	112–280	0.30	0.29	0.13	0.12
18	3- methylpentacosane	23.3	56–336	3.29	0.80	0.91	0.36
19	Hexacosane	23.5	366, 99, 85, 71, 57	1.93	0.47	0	
20	8-methylhexacosane	23.5	126–280	0.43	0.18	<0.01	
21	12,14 dimethylhexacosane	23.7	182–238, 196–210	0.27	0.09	0	
22	4-methylhexacosane	23.9	70–338	1.64	2.93	0	
23	2-methylhexacosane	24	350–56	0.83	0.15	0	
24	Heptacosene (I isomer)	24	378, 125, 111, 97, 83, 69, 55	0		6.53	2.62
25	Heptacosene (II isomer)	24.1	378, 125, 111, 97, 83, 69, 55				
26	Heptacosane	24.3	380, 99, 85, 71, 57	23.07	5.31	13.76	6.20
27	11- and 13-methylheptacosane	24.4	168–252 + 196–224	4.87	1.02	0.95	0.46
28	11,15 dimethylheptacosane	24.6	168, 196, 239, 266	11.40	2.85	0.84	0.29
29	3-methylheptacosane	24.7	56, 364	18.72	2.79	<0.01	
30	Octacosane	24.9	394, 99, 85, 71, 57	1.65	0.32	0	
31	3,7-dimethylheptacosane	25	56–378, 126–308	0.75	0.29	0	
32	12- and 14- and 16-methyloctacosane	25.1	182–253 + 210–224	0.73	0.22	<0.01	
33	12,16 dimethyloctacosane	25.3	182–266, 196–252	2.51	0.69	<0.01	
34	2-methyloctacosane	25.5	56–378	0.11	0.03	0	
35	Nonacosane	25.6	408, 99, 85, 71, 57	3.46	1.07	1.14	0.71
36	13- and 15-methylnonacosane	25.8	168–252 + 196–224	1.65	0.44	<0.01	
37	13,15 dimethylnonacosane	26	196–266, 224–238	6.29	2.05	<0.01	
38	3-methylnonacosane	26.3	56–302	1.41	0.32	<0.01	
40	Triacontane	28.1	420, 99, 85, 71, 57	0		1.12	0.76
41	Sterol	29.1	386	0		2.27	1.55

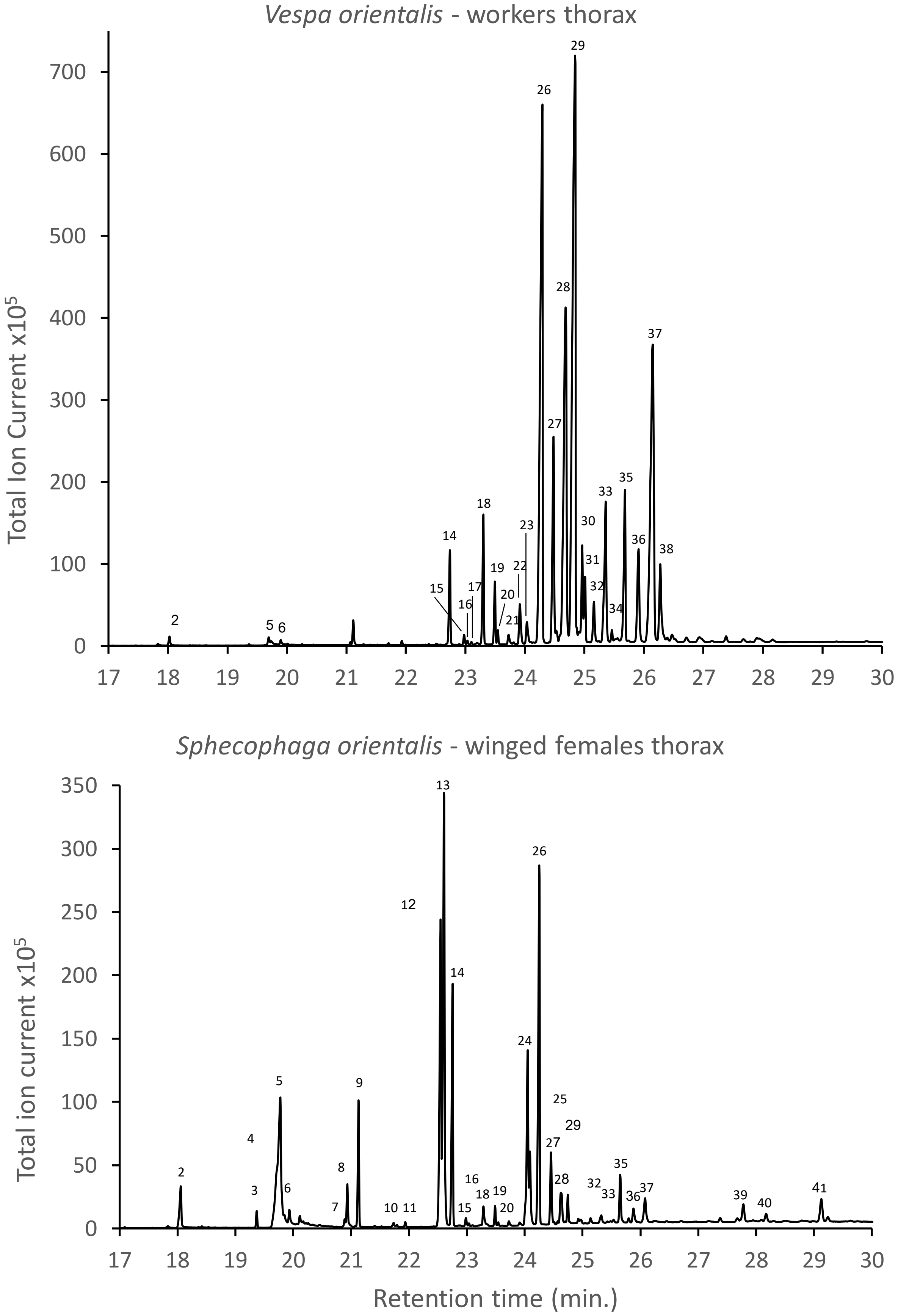

Vespa orientalis - workers thorax
Total Ion Current x10^5
0
100
200
300
400
500
600
700
17
18
19
20
21
22
23
24
25
26
27
28
29
30
2
5
6
14
15
16
17
18
19
20
21
22
23
26
27
28
29
30
31
32
33
34
35
36
37
38
Sphecophaga orientalis - winged females thorax
Total ion current x10^5
0
50
100
150
200
250
300
350
2
3
4
5
6
7
8
9
10
11
12
13
14
15
16
18
19
20
24
25
26
27
28
29
32
33
35
36
37
39
40
41
Retention time (min.)
